# Storm of soluble immune checkpoints associated with disease severity of COVID-19

**DOI:** 10.1038/s41392-020-00308-2

**Published:** 2020-09-07

**Authors:** Yaxian Kong, Yu Wang, Xueying Wu, Junyan Han, Guoli Li, Mingxi Hua, Kai Han, Henghui Zhang, Ang Li, Hui Zeng

**Affiliations:** 1 Institute of Infectious Diseases, Beijing Key Laboratory of Emerging Infectious Diseases, Beijing Ditan Hospital, Capital Medical University, Beijing, 100015 China; 2 Department of Respiratory Medicine, Beijing Ditan Hospital, Capital Medical University, Beijing, 100015 China; 3 Immupeutics Medicine Institute, Beijing, 100191 China; 4 Department of Intensive Care Medicine, Beijing Ditan Hospital, Capital Medical University, Beijing, 100015 China

**Keywords:** Infectious diseases, Infectious diseases, Adaptive immunity

**Dear Editor**,

As the outbreak of coronavirus disease 2019 (COVID-19) turns into a pandemic, it has literally caused a worldwide public health crisis. Progressive lymphopenia, especially in T cells, was a prominent clinical feature of severe COVID-19 in addition to dyspnea, hypoxemia, acute respiratory distress, and cytokine release syndrome.^1^ Recently, several studies revealed a correlation between T cell depletion and increased expression levels of several inhibitory checkpoint molecules on T cells in severe COVID-19 cases.^2^ Classically, inhibitory checkpoint molecules have been documented as key factors for regulating T cell exhaustion in a variety of chronic viral infections and tumors. Recent studies further implied a pivotal role of inhibitory checkpoint molecules in the pathophysiology of acute viral infections, such as Ebola virus or hantavirus infection. Of note, soluble isoforms of checkpoints can be produced by cleavage of membrane-bound proteins or by alternative splicing of mRNA and competitively regulate the functions of their membrane-bound counterparts.^3^ Thus it is of great interest to determine whether soluble checkpoint molecules are involved in immune regulation and severity of COVID-19.

To investigate the relationship between soluble checkpoint molecules and COVID-19 progression, we recruited a total of 109 patients with confirmed diagnosis of COVID-19 from Beijing Ditan Hospital. All baseline medical record information including clinical characteristics and laboratory data are shown in Table S1. The median age of the patients was 48 years (range 20–88) with 57.8% men and 42.2% women. Among these 109 patients, 5 (4.6%) were asymptomatic, 60 (55.0%) were mild or moderate (MM) cases, and 44 (40.4%) were severe or critical (SC) cases. Thirty-six patients (33.0%) had chronic diseases, including hypertension, diabetes, chronic pulmonary disease, chronic kidney disease, cardiovascular disease, hyperlipemia, and immune disorder. Consistent with previous reports, SC cases were characterized by older age and increased number of white blood cells and neutrophils, as well as significantly lower counts of total lymphocytes, CD3, CD4, and CD8 T cells, than MM cases. In addition, the serum concentrations of hemoglobin, D-dimer, C-reactive protein, lactate dehydrogenase, alanine aminotransferase, aspartate aminotransferase, and potassium were higher in SC patients than those in MM patients (Table S1).

We evaluated serum levels of 14 soluble checkpoints (sBTLA, sGITR, sHVEM, sIDO, sLAG-3, sPD-1, sPD-L1, sPD-L2, sTIM-3, sCD28, sCD80, s4-1BB, sCD27, and sCTLA-4) from all 109 COVID-19 patients within 3 days of the hospital admission. The serum levels of all tested molecules except for PD-L2 were significantly higher in the SC group than in the MM and asymptomatic groups (Fig. 1a and Supplementary Fig. 1). Dynamic analysis showed that 11 molecules (sGITR, s4-1BB, sTIM-3, sCD27, sLAG-3, sPD-1, sCD28, sCTLA-4, sBTLA, sHVEM, and sCD80) were persistently higher in SC patients than in MM cases during hospitalization (Supplementary Fig. 2). Consistent with the theory that soluble forms of checkpoint molecules are produced by cleavage of membrane-bound protein or by mRNA expression,^3^ we observed similar dynamic pattern of soluble and membrane-bound counterparts in six SC COVID-19 patients (Fig. 1b). In addition, flow cytometric analysis revealed greater levels of glucocorticoid-induced tumor necrosis factor receptor (GITR), 4-1BB, T cell immunoglobulin and mucin domain 3 (TIM-3), CD27, programmed cell death protein 1 (PD-1), and LAG-3 on CD4 and CD8 T cells from SC patients than in those from MM patients (Supplementary Fig. 3). Of note, the levels of eight soluble checkpoint molecules (sIDO, sGITR, s4-1BB, sTIM-3, sCD27, sLAG-3, sPD-1, and sCD28) were negatively correlated with absolute counts of total, CD4, and CD8 T cells but not neutrophil counts (Fig. 1c). We also measured 45 cytokines/chemokines/growth factors and identified significantly negative correlations between absolute counts of T cell subsets and levels of IP-10, interleukin (IL)-1RA, IL-6, GRO-alpha, IL-10, and IL-18 (Supplementary Fig. 4).

**Fig. 1:**
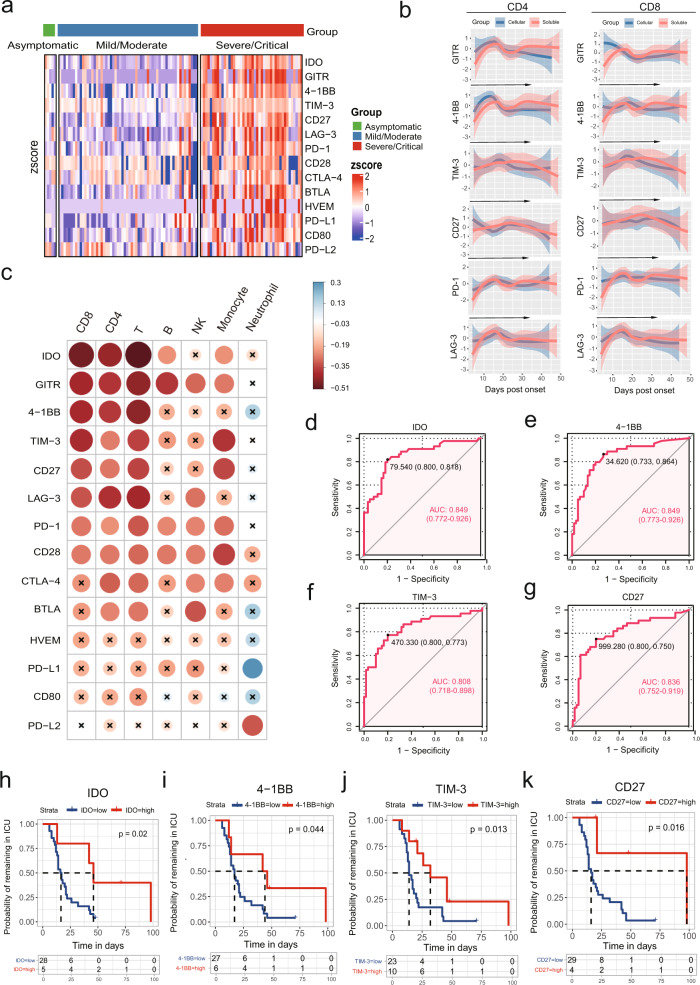
High level of soluble checkpoints correlated with severe illness in COVID-19 patients. **a** Heatmap depicting the relative serum levels of soluble checkpoint molecules in asymptomatic (*n* = 5), mild/moderate (*n* = 60), and severe/critical (*n* = 44) patients with COVID-19. Each column of the heatmap shows a patient, while the rows represent different molecules. Color scale in the heatmap represents scores standardized across rows. **b** The lines of best fit showing temporal changes of soluble and membrane-bound checkpoints in six severe/critical patients during hospitalization. The 95% confidence interval (CI) indicated by colored areas. **c** Correlation heatmap of peripheral blood leukocyte subsets and soluble checkpoints concentrations (data are log-transformed) at baseline. Data shown are representative of 72 patients with complete data. The circle size is proportional to the correlation coefficient value. Blue circle: positive correlation; red circle: negative correlation; values with no significant correlation are marked with a black cross. **d**–**g** Receiver operating characteristic (ROC) analyses for soluble IDO (**d**), 4-1BB (**e**), TIM-3 (**f**), and CD27 (**g**) in COVID-19 patients (*n* = 104). **h**–**k** Kaplan–Meier survival plots stratified by serum levels of soluble IDO (**h**), 4-1BB (**i**), TIM-3 (**j**), and CD27 (**k**). The 44 severe/critical patients contributed a total of 33 ICU admissions. Among ICU survivors, the proportion of patients remaining in the ICU is shown according to their ICU stay time

Next, we tested the predictive value of the candidate molecules in severity of COVID-19 (Table S2). Increased baseline levels of sIDO, s4-1BB, sTIM-3, and sCD27 were related to a higher disease severity rate, with area under the curve (AUC) values >0.8 (0.849 (95% confidence interval (CI) 0.772–0.926), 0.849 (95% CI 0.773–0.926), 0.808 (95% CI 0.718–0.898), and 0.836 (95% CI 0.752–0.919), respectively; Fig. 1d–g). When using an optimal cutoff value, patients with high baseline levels of sIDO, s4–1BB, sTIM-3, and sCD27 demonstrated a prolonged intensive care unit (ICU) stay time based on Kaplan–Meier (K-M) curves (*p* = 0.02, 0.04, 0.013, and 0.016, respectively; Fig. 1h–k). Compared to checkpoints, proinflammatory IL-6 also displayed a high AUC for predicting severity of COVID-19 (0.855 (95% CI 0.781–0.930)), but poor discrimination for ICU stay time (*p* = 0.098, Supplementary Fig. 5). In line with previous studies, IL-10, IP-10, and IL-18 also presented good predictive values for COVID-19 progression (AUC: 0.845 (95% CI 0.769–0.921), 0.838 (95% CI 0.758–0.917, and 0.823 (95% CI 0.743–0.903), respectively; K-M analysis: *p* = 0.029, *p* < 0.001, and *p* = 0.0043, respectively; Supplementary Fig. 5). Taken together, these soluble checkpoints were identified to have good predictive values as well as inflammatory cytokines in COVID-19 progression.

Here we provided evidence to link the storm of soluble immune checkpoints to COVID-19 progression. Consistently, a newly published study also observed elevation of sCD25, sTIM-3, sLAG-3, and sGalectin-9 in COVID-19 patients with active infection compared to patients after recovery.^4^ To date, the functions of most soluble molecules have not been fully addressed. The elevated levels of both membrane-bound and soluble checkpoint molecules reflect a broad and complicated dysregulation of T cell response in severe cases of COVID-19. In addition, immune checkpoints include stimulatory and inhibitory molecules that assist with immune response and maintain self-tolerance. Thus the overall effects of these heterogeneous checkpoint molecules on immune response are hardly computable. The depletion of T cells in COVID-19 patients might represent a result from the imbalance between membrane-bound and soluble molecules.

We further identified sIDO, s4-1BB, sTIM-3, and sCD27 as predictive biomarkers for disease severity of COVID-19. In agreement with our present findings, a recent clinical study found that treatment with immune checkpoint inhibitors (ICIs), mostly PD-1 or programmed death-ligand 1 (PD-L1) blockade, correlated with poor outcomes in COVID-19 patients with cancer.^5^ Given that sPD-1 or sPD-L1 could bind to the membrane-bound PD-L1 or PD-1 and consequently block the PD-1:PD-L1 pathway,^4^ intrinsic elevation of soluble checkpoints will have similar effects as extrinsic ICI therapy.

This study has several limitations. First, due to limited flow cytometric data acquired from a small number of patients, it is difficult to determine the association between soluble and cellular immune checkpoint molecules. Second, we conducted a single-center retrospective cohort study with small sample size of patients; more studies based on larger cohort in additional sites are necessary to verify our findings. Therefore, more evidences were urgently needed to investigate whether these soluble checkpoints might serve as potential therapeutic targets, with an aim to shedding new light on pathogenesis and treatment of COVID-19.

## Supplementary information

Supplementary Materials

## Data Availability

All data used to draw the conclusions in the paper are presented in the paper and/or Supplementary Materials.
