# Supplementary material for: Storm of soluble immune checkpoints associated with disease severity of COVID-19
Source: Signal Transduct Target Ther. 2020 Sep 7;5:192. doi: 10.1038/s41392-020-00308-2 (PMC7475713; doi:10.1038/s41392-020-00308-2)
Supplement: Supplementary file 1 — Supplementary Materials [file 41392_2020_308_MOESM1_ESM.pdf]

Supplementary Materials for

Storm of Soluble Immune Checkpoints Associated with Disease Severity of COVID-19

Yaxian Kong[1#], Yu Wang[2#], Xueying Wu[3#], Junyan Han[1], Guoli Li[1], Mingxi Hua[1], Kai Han[1], Henghui Zhang[1*], Ang Li[4*], Hui Zeng[1*]

[1]Institute of Infectious Diseases, Beijing Key Laboratory of Emerging Infectious Diseases, Beijing Ditan Hospital, Capital Medical University, Beijing 100015, China

[2]Department of Respiratory Medicine, Beijing Ditan Hospital, Capital Medical University, Beijing 100015, China

[3]Immupeutics Medicine Institute, Beijing 100191, China

[4]Department of Intensive Care Medicine, Beijing Ditan Hospital, Capital Medical University, Beijing 100015, China

[#]These authors contributed equally to this work

***Correspondence to:**

Hui Zeng (zenghui@ccmu.edu.cn), Ang Li (liang@ccmu.edu.cn), Henghui Zhang (zhhbao@ccmu.edu.cn)

This PDF file includes:

Materials and Methods

Figures. S1 to S5

Tables S1 to S2

Materials and Methods

Patients

A total of 109 COVID-19 patients in this retrospective cohort study were enrolled from Beijing Ditan Hospital from January 20, 2020 to March 27, 2020. All enrolled patients were confirmed to be infected with SARS-CoV-2 by RT-PCR assays on pharyngeal swab specimens. This study was approved by Committee of Ethics at Beijing Ditan Hospital, Capital Medical University with informed consents acquired from all enrolled patients. The severity of COVID-19 was defined as asymptomatic, mild, moderate, severe and critical, according to the guidelines on the diagnosis and treatment of new coronavirus pneumonia (version 7) by the National Health Commission of China issued on 3 March 2020.

Briefly, asymptomatic disease was defined as normal body temperature, lack of respiratory symptoms and no pulmonary radiological manifestation. Mild cases had minor symptoms and no radiological evidence of pneumonia. Moderate disease was defined as having fever, respiratory symptoms and radiological evidence of pneumonia. Severe cases were diagnosed based on one of the following criteria: respiratory rate > 30/min, oxygen saturation levels (SpO2) <93%, arterial partial pressure of oxygen (PaO2)/ fraction of inspired oxygen (FiO2)(PaO2/ FiO2 ratio) <=300mmHg or pulmonary imaging shows multi-lobular lesions or lesion progression exceeding 50% within 48 hours. Critical disease was defined as one of the following: acute respiratory distress syndrome requiring mechanical ventilation, shock, complications with other organ failure. These 109 patients included 5 (4.6%) asymptomatic patients, 60 (55.0%) mild or moderate (MM) patients, and 44 (40.4%) severe or critical (SC) patients.

All baseline medical record information including demographic data and clinical characteristics were obtained within the first day after hospital admission (Table S1). Blood samples were first collected within three days of the hospital admission and once every 3-7 days during hospitalization. The median age of the patients was 48 years (range 20-88) with 57.8% men and 42.2% women. Among these 109 patients, 36 (33.0%) had at least one comorbidity, the most common were hypertension (24 cases),

diabetes (13 cases), chronic pulmonary disease (8 cases), chronic kidney disease (5 cases), cardiovascular disease (4 cases), hyperlipemia (3 cases), and immune disorder (2 cases).

Serum multiplexed bead immunoassays

We tested serum proteins consisting of two parts: part I, ProcartaPlex Human Immuno-Oncology Checkpoint Panel (Invitrogen, Calsbad, CA, USA), including BTLA, GITR, HVEM, IDO, LAG-3: 47, PD-1, PD-L1, PD-L2, TIM-3, CD28, CD80, 4-1BB, CD27,and CD152; part II, ProcartaPlex Human Cytokine/Chemokine/Growth Factor Panel (Invitrogen), including BDNF, Eotaxin/CCL11, EGF, FGF-2, GM-CSF, GROα/CXCL1, HGF, NGFβ, LIF, IFNα, IFNγ, IL-1β, IL-1α, IL-1Rα, IL-2, IL-4, IL-5, IL-6, IL-7, IL-8/CXCL8, IL-9, IL-10, IL-12p70, IL-13, IL-15, IL-17α, IL-18, IL-21, IL-22, IL-23, IL-27, IL-31, IP-10/CXCL10, MCP-1/CCL2, MIP-1α/CCL3, MIP-1β/CCL4, RANTES/CCL5, SDF-1α/CXCL12, TNFα, TNFβ/LTA, PDGF-BB, PLGF, SCF, VEGF-A, and VEGF-D. Data were acquired according to manufacturer’s protocol using Luminex MAGPIX® instrument (Luminex Co., Austin, TX, USA) and analyzed using ProcartaPlex Analyst 1.0 software (Invitrogen).

Immunofluorescence staining and flow cytometric analysis

Peripheral blood mononuclear cells (PBMCs) were incubated with directly conjugated antibodies for 30 minutes at 4°C. The cells were then washed before flow cytometric analysis. Antibodies used were anti-human CD3-BV786, CD8-BV510, PD-1-BV711, 4-1BB-BV421, GITR-BV605, CD27 PE (BD Biosciences, San Diego, CA, USA), CD4-APC-Fire750, TIM-3-FITC, LAG-3-APC (BioLegend, San Diego, CA, USA) antibodies, and corresponding isotype controls. Data were acquired on a LSR Fortessa flow cytometer (BD Biosciences) and analyzed using FlowJo Software (Tree Star, Ashland, OR, USA).

Statistical analysis

Quantitative variables are presented as means and SDs or medians and interquartile

ranges, and categorical variables are presented as proportions. The t test was used to analyze difference between 2 groups of normally distributed variables, and the Mann-Whitney U test was used for nonnormally distributed variables. The Fisher's exact test was used to compare categorical variables. The correlations between serum protein levels and different variables were analyzed by Spearman rank correlation test. A receiver operating characteristic (ROC) curve was generated to evaluate the diagnostic accuracy of a protein. The area under the ROC curve (AUC) was used as a measure of discriminatory ability for the signature scores. Survival analysis was performed using a Kaplan–Meier survival plot, and the log-rank test P value was calculated. The 'surv_cutpoint' function of the 'survminer' R package was used to iteratively determine the optimal cutpoints of continuous variables achieving the maximally selected rank statistics. All statistical analyses were performed using R version 3.6.3 software (Institute for Statistics and Mathematics, Vienna, Austria; www.r-project.org). $P < 0.05$ was considered statistically significant, and tests were 2-tailed.

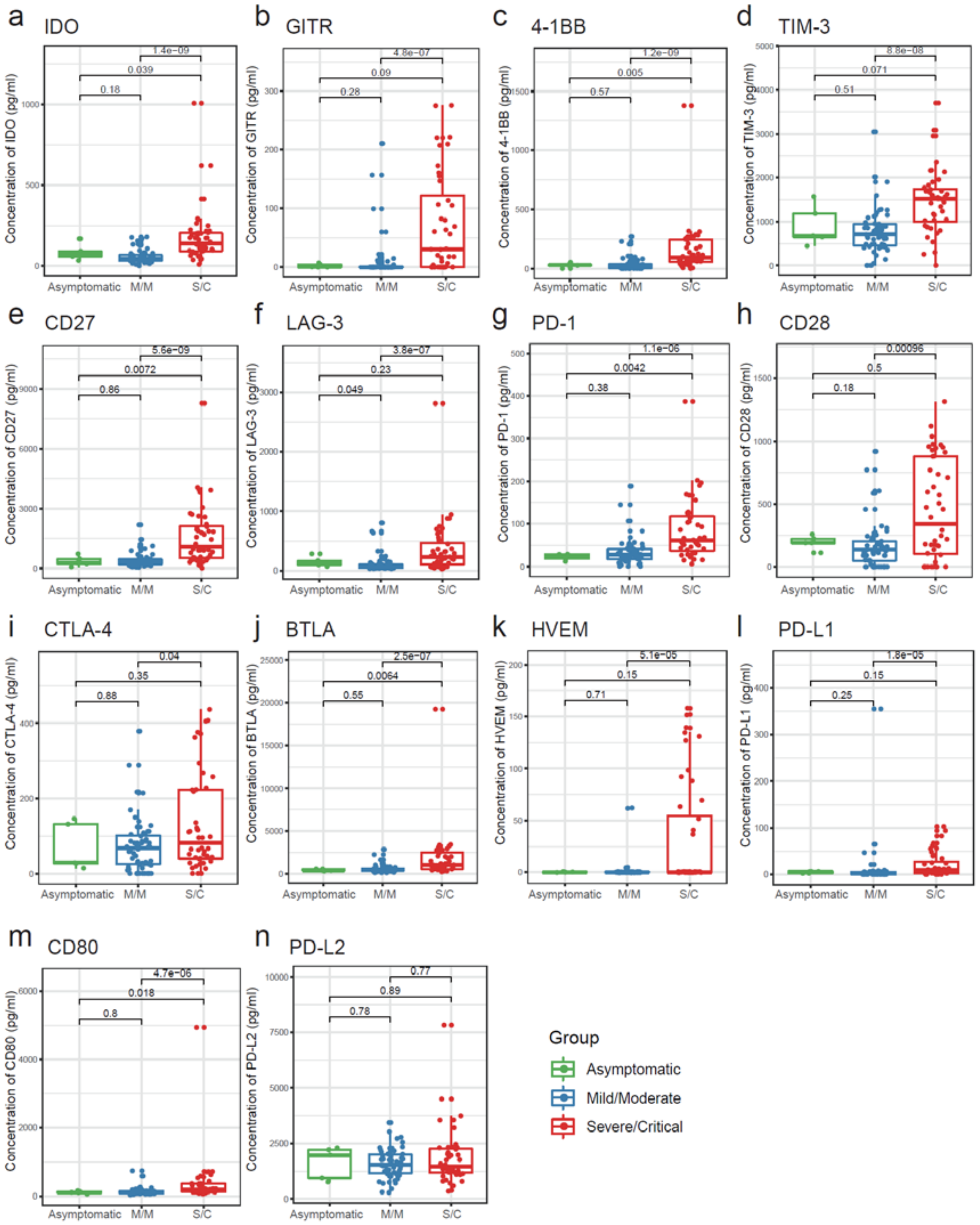

Figure S1. Comparisons of soluble checkpoints concentrations between groups. Boxplots display the serum levels of 14 soluble checkpoints in asymptomatic (n =5), mild/moderate (n = 60) and severe/critical (n = 44) patients. *P*-values were calculated with the Wilcoxon test; the box shows the upper and lower quartiles. M/M: mild/moderate; S/C: severe/critical.

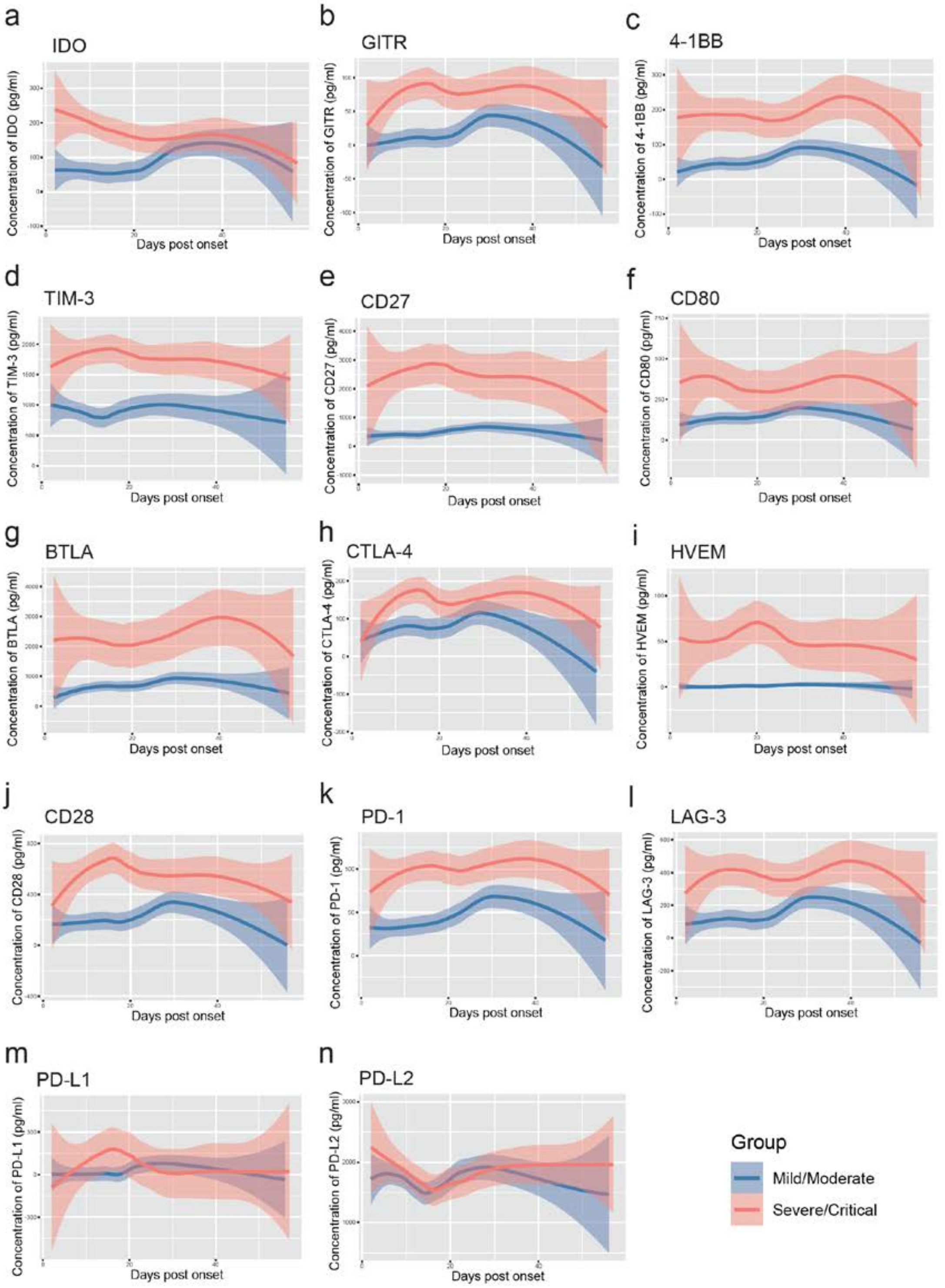

Figure S2. Dynamic profiles of levels of soluble checkpoints in COVID-19 patients. The lines of best fit showing temporal changes of 14 soluble checkpoints in mild/moderate (n = 60) and severe/critical (n = 44) patients during hospitalization. The 95% confidence interval (CI) indicated by colored areas.

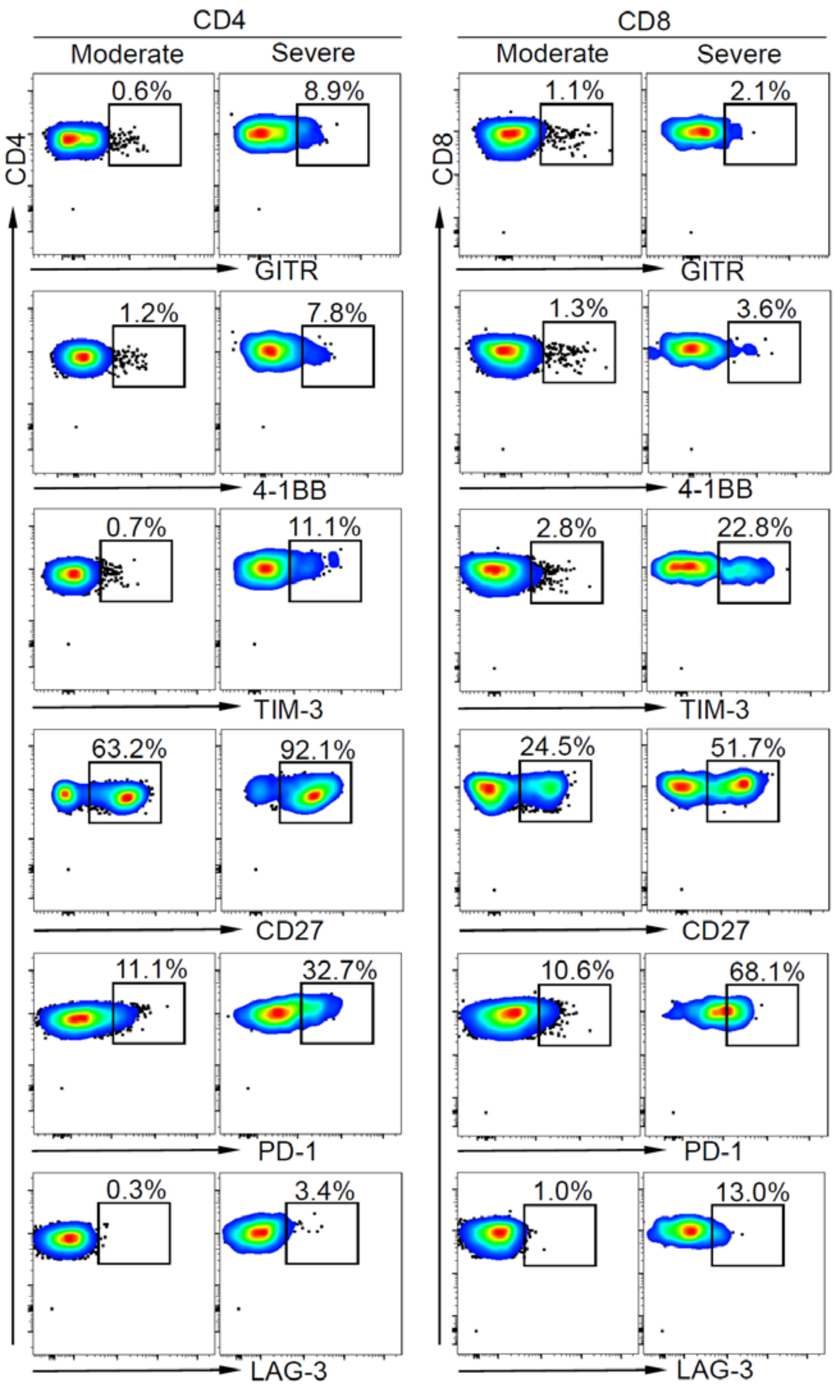

Figure S3. Expression of membrane-bound checkpoints on T cells in COVID-19 patients. Representative flow data of expression of GITR, 4-1BB, TIM-3, CD27, PD-1, and LAG-3 on CD4 and CD8 T cells.

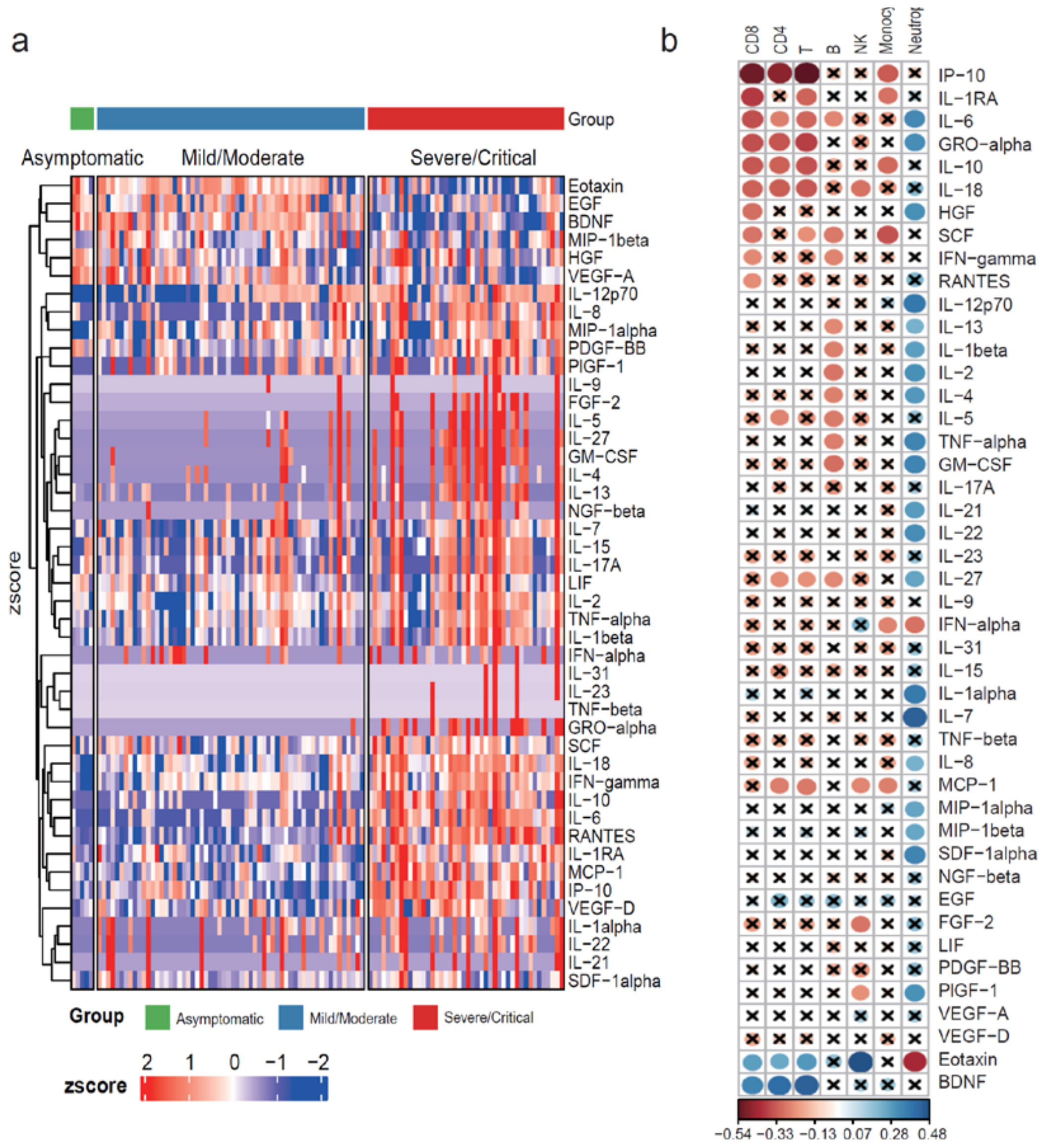

Figure S4. The relationship of serum levels of cytokines and peripheral blood leukocyte subsets in COVID-19 patients. a, Heatmap depicting the relative cytokines concentrations in asymptomatic (n =5), mild/moderate (n = 60), and severe/critical (n = 44) patients. Each column of the heatmap shows a patient, while the rows represent different cytokines. Color scale in the heatmap represents scores standardized across rows. **b**, Correlation heatmap of peripheral blood leukocyte subsets and 45 cytokines concentrations (data are log-transformed) at baseline. Data shown are representative of 73 patients with complete data. *P* and correlation coefficient values were obtained using the Spearman's correlation test. The circle size is proportional to the correlation coefficient value. Blue circle: positive correlation; red circle: negative correlation; values with no significant correlation are marked with a black cross.

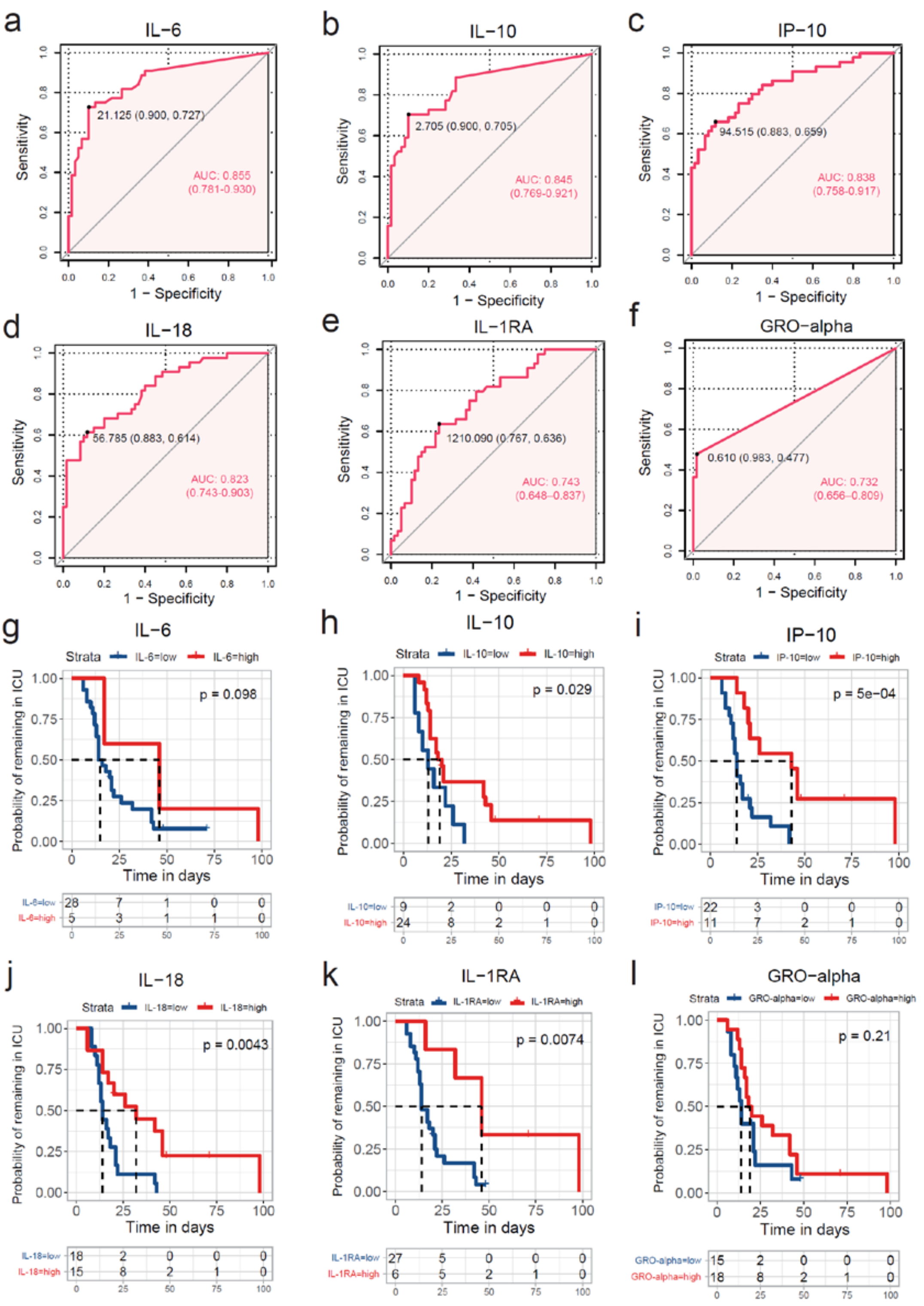

Figure S5. Predictive value of candidate cytokines for COVID-19 progression. a-f, Receiver operating characteristic (ROC) curves showing the sensitivity and specificity for IL-6 (a), IL-10 (b), IP-10 (c), IL-18 (d), IL-1RA (e) and GRO-alpha (f) cutoff values on patients' classification. Area under the curve (AUC) values and 95% confidence

interval (CI) are presented. **g-l**, Kaplan-Meier survival plots stratified by concentration of IL-6 (g), IL-10 (h), IP-10 (i), IL-18 (j), IL-1RA (k) and GRO-alpha (l). *P*-values were obtained by the log-rank test. The 44 severe/critical patients contributed a total of 33 ICU admissions. Among ICU survivors, the proportion of patients remaining in the ICU is shown according to their ICU stay time.

Table S1. Demographics, baseline **c**haracteristics of COVID-19 patients

Characteristics	Total (n = 109)	Asymptomatic patients (n=5, Group1)	Mild/Moderate patients (n = 60, Group2)	Severe/Critical patients (n = 44, Group3)	P values (Group 1 *vs* 2)	P values (Group 1 *vs* 3)	P values (Group 2 *vs* 3)
Age mean range, y	48 (20, 88)	43 (21, 58)	39 (20, 76)	61 (20, 88)	0.43	0.033	<0.001
Gender					1	1	0.654
Male n (%)	63 (57.8)	3 (60.0)	33 (55.0)	27 (61.4)			
Female n (%)	46 (42.2)	2 (40.0)	27 (45.0)	17 (38.6)			
Any Comorbidity, n (%)	36 (33.0)	2 (40.0)	4 (6.7)	30 (68.2)	0.095	0.448	<0.001
Hypertension	24 (22.0)	2 (40.0)	3 (5.0)	19 (43.2)	0.051	1	<0.001
Cardiovascular disease	4 (3.7)	0 (0.0)	0 (0.0)	4 (9.1)	NA	1	0.062
Chronic Pulmonary disease	8 (7.3)	0 (0.0)	0 (0.0)	8 (18.2)	NA	0.686	0.002
Diabetes	13 (11.9)	1 (20.0)	0 (0.0)	12 (27.3)	0.11	1	<0.001
Hyperlipemia	3 (2.8)	0 (0.0)	2 (3.3)	1 (2.3)	1	1	1
Chronic kidney disease	5 (4.6)	0 (0.0)	1 (1.7)	4 (9.1)	1	1	0.199
Immune disorders	3 (2.8)	0 (0.0)	0 (0.0)	3 (6.8)	NA	1	0.144
Others	1 (0.9)	0 (0.0)	0 (0.0)	1 (2.3)	NA	1	0.876
Laboratory data, mean (SD)							
WBC, 10^9/L	6.07 (3.21)	4.86 (1.33)	5.22 (1.69)	7.30 (4.32)	0.642	0.218	0.001
Lymphocyte, 10^9/L	1.46 (0.70)	2.18 (0.87)	1.67 (0.69)	1.10 (0.50)	0.122	<0.001	<0.001
Neutrophil, 10^9/L	4.22 (3.17)	2.21 (0.89)	3.14 (1.31)	5.85 (4.18)	0.131	0.061	<0.001
Monocyte, 10^9/L	0.33 (0.14)	0.40 (0.09)	0.33 (0.12)	0.31 (0.17)	0.19	0.283	0.656
$CD3^+$ T cells/μl	982.84 (510.65)	1541.25 (756.57)	1188.00 (436.80)	673.05 (389.79)	0.148	<0.001	<0.001
$CD4^+$ T cells/μl	557.71 (282.84)	830.50 (499.21)	634.58 (238.02)	435.13 (262.68)	0.156	0.012	<0.001
$CD8^+$ T cells/μl	389.29 (268.76)	657.25 (294.69)	504.85 (260.32)	219.56 (162.49)	0.27	<0.001	<0.001
B cells/μl	192.64 (106.35)	168.00 (123.11)	199.17 (112.26)	183.96 (94.54)	0.599	0.766	0.572

NK cells/μl	206.08 (137.92)	227.00 (196.17)	222.74 (131.96)	169.96 (139.09)	0.953	0.478	0.122
Hemoglobin, g/L	134.09 (18.52)	142.40 (12.56)	141.23 (16.52)	123.89 (16.90)	0.878	0.022	<0.001
Platelets, 10^9/L	231.52 (92.55)	262.40 (71.53)	237.48 (77.41)	220.29 (111.13)	0.491	0.414	0.362
PT, s	12.67 (3.36)	11.12 (0.87)	11.85 (0.84)	13.64 (4.63)	0.109	0.29	0.022
APTT, s	31.83 (5.41)	29.30 (1.94)	31.95 (3.26)	31.98 (7.13)	0.121	0.462	0.979
D-dimer, mg/L	1.63 (4.30)	0.13 (0.09)	0.38 (0.37)	3.04 (5.96)	0.189	0.339	0.007
CRP, mg/L	30.43 (55.48)	2.40 (1.71)	8.87 (12.14)	60.57 (74.65)	0.243	0.091	<0.001
CK, U/L	95.79 (101.30)	75.56 (31.62)	90.78 (82.71)	105.57 (128.57)	0.686	0.609	0.504
LDH, U/L	257.62 (123.32)	170.73 (31.00)	203.93 (54.96)	343.92 (148.68)	0.24	0.027	<0.001
ALT, U/L	34.33 (36.29)	27.46 (7.47)	28.22 (22.81)	43.14 (49.18)	0.941	0.484	0.05
AST, U/L	28.99 (17.81)	19.82 (5.60)	23.89 (12.99)	36.79 (21.21)	0.539	0.122	<0.001
TBIL, mmol/L	13.48 (12.47)	12.28 (2.34)	11.56 (6.87)	16.19 (17.60)	0.838	0.662	0.081
Albumin, g/L	40.07 (5.66)	44.92 (3.97)	43.01 (3.73)	35.79 (5.02)	0.281	<0.001	<0.001
sCr, mmol/L	78.80 (78.35)	66.34 (25.29)	73.45 (25.93)	87.03 (117.48)	0.558	0.699	0.403
K^+, mmol/L	3.96 (0.40)	3.77 (0.18)	3.89 (0.38)	4.08 (0.42)	0.475	0.108	0.02
Na^+, mmol/L	139.20 (3.63)	140.84 (1.70)	139.71 (2.16)	138.34 (4.94)	0.263	0.27	0.062

WBC: white blood cells; NK: natural killer, PT: prothrombin time; APTT: activated partial thromboplastin time; CRP: C-reactive protein; CK, creatine kinase; LDH: lactate dehydrogenase; ALT: alanine aminotransferase; AST: aspartate aminotransferase; TBIL: total bilirubin; sCr: serum creatinine.

Table S2. The ROC analysis of variables associated with the severity of COVID-19 (n=104[a])

Variables	AUC	95% CI	Cut-off value	Youden's Index	Sensitivity (%)	Specificity (%)
IDO	0.849	(0.772-0.926)	79.540	0.618	81.8	80
4-1BB	0.849	(0.773-0.926)	34.620	0.597	86.4	73.3
CD27	0.836	(0.752-0.919)	470.330	0.573	77.3	80
TIM-3	0.808	(0.718-0.898)	999.280	0.550	75	80
BTLA	0.797	(0.707-0.887)	759.495	0.544	72.7	81.7
LAG-3	0.792	(0.698-0.886)	148.595	0.565	68.2	88.3
PD-1	0.78	(0.687-0.874)	42.465	0.488	70.5	78.3
CD80	0.764	(0.67-0.857)	158.780	0.442	65.9	78.3
GITR	0.762	(0.672-0.852)	15.265	0.514	61.4	90
PD-L1	0.746	(0.643-0.848)	7.030	0.519	63.6	88.3
CD28	0.689	(0.577-0.802)	358.420	0.417	50	91.7
HVEM	0.647	(0.573-0.72)	20.645	0.301	31.8	98.3
CTLA-4	0.619	(0.507-0.731)	218.025	0.262	29.5	96.7
PD-L2	0.483	(0.366-0.599)	1430.745	0.117	50	61.7

ROC: receiver operating characteristic; AUC: area under curve; CI: confidence interval; [a]5 asymptomatic patients were excluded from the ROC analysis.
